# Muscle strength and bone density in patients with different rheumatic conditions: cross-sectional study

**DOI:** 10.3325/cmj.2011.52.164

**Published:** 2011-04

**Authors:** Selma Cvijetić, Simeon Grazio, Milica Gomzi, Ladislav Krapac, Tomislav Nemčić, Melita Uremović, Jasminka Bobić

**Affiliations:** 1 Department for Environmental and Occupational Health, Institute for Medical Research and Occupational Health, Zagreb, Croatia; 2 Department of Rheumatology, Physical and Rehabilitation Medicine, Sisters of Mercy University Hospital, Zagreb, Croatia; 3 Drago Čop Polyclinics for Rheumatology, Physical Medicine and Rehabilitation, Zagreb, Croatia; 4 Domnius Polyclinic for Physical Medicine and Rehabilitation, Zagreb, Croatia

## Abstract

**Aim:**

To explore the relationship between muscle strength and bone density in patients with different rheumatic diseases and to examine whether inflammatory arthritis was more harmful for muscle strength and bone loss than degenerative joint diseases.

**Methods:**

The study included 361 men and women with a mean ± standard deviation age of 60.5 ± 11.4 years and different rheumatic conditions: regional syndromes, osteoarthritis of the hands, shoulders, knees, and hips, and inflammatory arthritis. Maximum voluntary back strength was measured by isometric dynamometry. Bone mineral density (BMD; g/cm^2^) of the lumbar spine, femoral neck, and distal radius was measured by dual-energy x-ray absorptiometry. Anthropometry and lifestyle characteristics were also assessed.

**Results:**

Back strength was lowest in patients with hand and shoulder osteoarthritis (20.0 ± 17.9 kg), followed by patients with inflammatory arthritis (24.8 ± 19.2 kg). Patients with inflammatory arthritis had the lowest BMD at the mid-radius (0.650 ± 0.115 g/cm^2^) and femoral neck (0.873 ± 0.137 g/cm^2^), while patients with hand and shoulder osteoarthritis had the lowest BMD at the mid-radius (0.660 ± 0.101). In both sexes, muscle strength was significantly lower in patients who had lower BMD (T score<-1.0). Multiple regression analysis identified significant predictors of back strength to be spine BMD (*P* = 0.024) and body mass index (*P* = 0.004) in men and femoral neck BMD in women (*P* = 0.004).

**Conclusion:**

Muscle strength decline may be connected to bone loss in patients with rheumatic conditions, especially those with inflammatory joint diseases.

There is a concomitant decline in muscle strength of the upper and lower limbs and bone density after the fifth decade of the life (1,2). Impaired muscle function is a common consequence in patients with rheumatic diseases, especially those with inflammatory joint diseases. Muscle strength may also be significantly reduced around joints affected with osteoarthritis. Several studies showed greatly reduced isokinetic strength in patients with rheumatoid arthritis (3-5) and patients with knee osteoarthritis (6).

It is also known that muscle strengthening can yield a bone-building effect (7). Exercises with greater loading and higher impact activities produce the greatest skeletal benefit (8). Increased muscle weakness can also compound the problem of low bone density by increasing the risk of falls and fracture. A positive correlation between muscle strength and bone density has been shown in several studies (9-17). Some of them demonstrated the association only in postmenopausal women (12,17) but not in men (9,13), while other found a site-specific correlation between muscle strength and bone mineral density (BMD) (4,12). However, several studies did not find a correlation between any measures of muscle strength and BMD (18,19). With such contradictory reports, it is difficult to make clinically relevant conclusions about the relationship between muscle strength and bone mass, although this may be one of the key factors that affect the rehabilitation outcome.

The aim of the study was to assess the differences in muscle strength and bone density between patients with different rheumatic conditions. Since muscle strength is an important determinant of bone density, we explored whether the age-related decline in bone density and muscle strength was more pronounced in patients with inflammatory arthritis than in those with degenerative joint diseases.

## Materials and methods

### Participants

The study included 361 patients referred to specialists in physical medicine, with subspecialization in rheumatology (rheumatologists) from the Department of Rheumatology, Physical and Rehabilitation Medicine, Sisters of Mercy University Hospital; Drago Čop Polyclinics for Rheumatology, Physical Medicine, and Rehabilitation; and Domnius Polyclinic for Physical Medicine and Rehabilitation due to rheumatic complaints in the period between 2006 and 2009. The patients had the following diagnoses: regional syndromes (cervicobrachial syndrome, lumbosacral syndrome) (n = 226), osteoarthritis of the hands and shoulders (n = 26), osteoarthritis of the knees and hip (n = 60), and inflammatory arthritis (non-specified polyarthritis, rheumatoid arthritis, ankylosing spondylitis) (n = 36). Anthropometric measurements (height, weight, body mass index) and back dynamometry (in pounds) were performed in all patients. Information on lifestyle habits, including smoking and drugs consumption, were obtained using an interviewer-administered questionnaire designed for this study. The smoking index was calculated by multiplying the number of cigarettes with the years of smoking.

The study was approved by the Ethics Committee of the Institute for Medical Research and Occupational Health. All participants signed an informed consent.

### Anthropometry, dynamometry, and blood pressure measurement

Height and weight were measured using a portable stadiometer and electronic scale. Body mass index was calculated as weight (kg) divided by the squared height (m^2^). The maximum voluntary back strength was recorded isometrically using a Lafayette-Adult Back and Leg Dynamometer Package Model 32527 A-3 (Lafayette Instrument Company, Lafayette, IN, USA). The package includes a 600-pound pull dynamometer, foot chain, solid aluminum lifting bar with comfortable hand grips, and a lifting platform. The solid lifting platform, measuring 61 cm ×61 cm, is small enough for easy transportation. The pull dynamometer has several heavy-duty springs for long-lasting accuracy and a range of 50 to 600 pounds, in 5-pound increments. The back dynamometer has an adjustable handgrip chain and a scale for quick and reliable reading. Before a measurement, the technician explained and demonstrated the correct exercise method to the participant. The chain was adjusted so that the gripping bar met the knee height of the participant at the measured posture. For back strength measurement, the participant stood on the platform with his/her feet a suitable distance apart (about shoulder width), grasped the gripping bar at both ends, kept the leg straight, bent the body forward around the hip joint (the chest forward and the head erect), and extended his/her trunk around the hip joint by pulling the bar upwards to the maximum voluntary force (at the same time, the trunk reached its final angle), and then the force value was recorded. Each strength item was measured two times for each participant, and the mean of two readings was used as the result. The precision error of the measurement was 3.8%. Original measurement units of the system are pounds but are here presented in kilograms.

Blood pressure was measured in a sitting position by a mercury sphyngo-manometer (Reister Co., Jungingen, Germany).

### Bone density measurement

Bone mineral density (BMD; g/cm^2^) at the lumbar spine, femoral neck, total hip, and distal third of radius (mid-radius) was measured using dual-energy x-ray absorptiometry (Prodigy, GE Healthcare, Madison, WI, USA). The in-vivo coefficient of variation was 1.5% for the lumbar spine, 1.1% for the femoral neck, 2.1% for the total hip, and 2.2% for the distal radius. BMD was also expressed as T score, which represents the number of standard deviations with respect to the mean BMD of a control population between 20 and 40 years, using the manufacturer’s reference values.

### Statistics

Data were analyzed using the software Statistica, version 9.0 (StatSoft Inc., Tulsa, OK, USA). The results are shown as mean ± standard deviation for normally distributed data or as median and interquartile range for variables that did not show normal distribution (back strength and smoking index). Differences in the means between men and women, pre- and post-menopausal women, patients with shoulder osteoarthritis and patients with all other rheumatic diagnoses, and patients with lower and higher BMD were tested using *t* test. The relationship between two variables was tested with the linear correlation. Analysis of co-variance (ACNOVA) was used to test the differences in dynamometry and bone density between different rheumatic groups of patients, after removing the variance for which covariates (age, sex, and body mass index) account. The multiple regression model was created with BMD as a dependent variable and age, body mass index, muscle strength, smoking, and steroid/thyroxine therapy as independent variables. The normality of distribution of variables was tested using the Kolmogorov-Smirnov test. The variables that were not distributed normally (back strength and smoking index) were recalculated to the new variables using the logarithmic function, and the log-transformed variables were used in *t* test, ANCOVA, and multiple regression. In all tests, *P* value lower than 0.05 was considered significant.

## Results

There were 257 women with mean age of 59.4 ± 11.5 years and 104 men with mean age of 61.6 ± 11.3 years (*P* = 0.107). Forty four women were pre-menopausal (17.1%). Thirty four women (13%) and 7 men (7%) were taking drugs for osteoporosis and osteopenia (estrogen, bisphosphonates, or raloxifen), 4 women and 5 men were taking glucocorticoid therapy, while 18 women and 1 man were taking thyroxin replacement therapy. Also, 41 women (16%) and 5 men (5%) were on calcium and vitamin D.

Men had significantly higher dynamometry values than women (Table 1). Women had significantly lower BMD at the spine and mid-radius, whereas men had significantly higher smoking index (Table 1).

**Table 1:** Characteristics of participants with different rheumatic conditions (mean ± standard deviation)

Characteristic	Women (n = 257)	Men (n = 104)	P*
Age (years)	59.4 ± 11.5	61.6 ± 11.3	0.107
Height (cm)	162.1 ± 6.3	172.9 ± 7.7	<0.001
Weight (kg)	70.8 ± 12.8	79.9 ± 14.7	<0.001
Body mass index (kg/m^2^)	26.9 ± 4.5	26.9 ± 3.9	0.527
Back strength (kg)^†^	28.3 ± 23.6	48.1 ± 33.9	<0.001
Smoking index^†‡^	391.3 ± 308.3	771.3 ± 376.8	<0.001
Spine (g/cm^2^)	1.088 ± 0.186	1.162 ± 0.380	0.014
Femoral neck bone mineral density (g/cm^2^)	0.887 ± 0.124	0.929 ± 0.409	0.144
Total hip bone mineral density (g/cm^2^)	0.940 ± 0.136	0.990 ± 0.416	0.087
Mid-radius bone mineral density (g/cm^2^)	0.618 ± 0.108	0.713 ± 0.316	<0.001

*t test.

†Log-transformed before testing.

‡Calculated by multiplying the number of cigarettes with the years of smoking.

Patients with inflammatory arthritis (n = 26) had the lowest BMD at the spine and femoral neck (Table 2), while patients with hand and shoulder osteoarthritis (n = 36) had the lowest BMD at the radius. However, the difference in BMD between patients with different rheumatic diagnosis, when controlling for sex, age, and BMI (covariates), was not significant (spine: F = 0.139, *P* = 0.967, df = 4; femoral neck: F = 0.011, *P* = 0.955, df = 4; total hip: F = 0.021, *P* = 0.885, df = 4; mid-radius: F = 0.016, *P* = 0.313, df = 4). BMD in women significantly correlated with age (r = -0.17, *P* = 0.025 for spine; r = -0.29, *P* < 0.001 for neck; r = -0.28, *P* < 0.001 for mid-radius) and with body mass index (r = 0.21, *P* = 0.007 for neck; r = 0.18, *P* = 0.021 for mid-radius). In men, BMD significantly correlated with body mass index (r = 0.32, *P* = 0.010 for spine; r = 0.26, *P* = 0.037 for neck; r = 0.28, *P* = 0.026 for mid-radius).

**Table 2:** Bone mineral density (BMD) and back strength in patients according to their rheumatic diagnosis (mean ± standard deviation)

	BMD (g/cm^2^)				Back strength (kg)
Rheumatic diagnosis	spine	femoral neck	total hip	mid-radius	
Cervicobrachial syndrome (n = 81)	1.115 ± 0.197	0.889 ± 0.135	0.966 ± 0.116	0.633 ± 0.093	31.3 ± 28.1
Lumbosacral syndrome (n = 145)	1.108 ± 0.332	0.906 ± 0.339	0.944 ± 0.392	0.643 ± 0.271	35.6 ± 27.5
Hand and shoulder osteoarthritis (n = 26)	1.160 ± 0.196	0.898 ± 0.117	0.937 ± 0.176	0.660 ± 0.101	20.0 ± 17.9
Knee and hip osteoarthritis (n = 60)	1.134 ± 0.177	0.923 ± 0.151	0.983 ± 0.153	0.665 ± 0.108	34.4 ± 27.1
Inflammatory arthritis (n = 36)	1.099 ± 0.178	0.873 ± 0.137	0.904 ± 0.127	0.650 ± 0.115	24.8 ± 19.2

**Figure 1:**
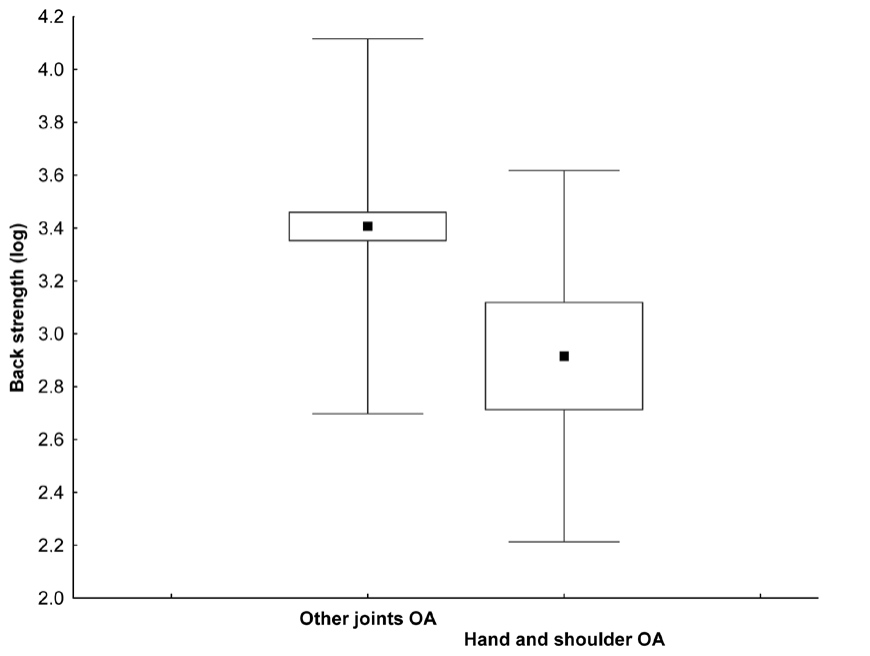
Back strength in patients with hand and shoulder osteoarthritis (OA) and in patients with osteoarthritis of the other joints. Square – mean; box – standard error; whisker – standard deviation. *t* = 2.32; *P* = 0.021 (*t* test).

Back strength was lowest in patients with hand and shoulder osteoarthritis, followed by patients with inflammatory arthritis (Table 2). There was no significant between-group difference in dynamometry according to the rheumatic diagnosis, when controlling for sex, age, and BMI (covariates) (F = 2.236, *P* = 0.066, df = 4). Patients with hand and shoulder osteoarthritis had significantly lower muscle strength than patients with all other rheumatic diagnoses (Figure 1). In women, muscle strength significantly correlated with age (r = -0.19; *P* = 0.010) and neck BMD (r = 0.16, *P* = 0.027). In men, dynamometry values significantly correlated only with BMI (r = 0.37; *P* = 0.021). Postmenopausal women had lower muscle strength (60.7 ± 49.7) than pre-menopausal women (70.9 ± 57.4), but the difference was not significant (*P* = 0.229). Muscle strength was significantly lower in male patients with lower BMD (T score≤-1.0) at all measured regions than in patients with normal BMD (T score>-1.0) (Table 3). In women, muscle strength was significantly lower in patients with lower BMD at the spine and mid-radius.

**Table 3:** Back strength (kg) in patients according to their T score at the spine, femoral neck, total hip, and mid-radius (mean ± standard deviation)*

Region	T score>-1.0		T score≤-1.0	
	women	men	women	men
Spine	27.4 ± 23.1 (n = 106)^†^	56.5 ± 36.6 (n = 35)^‡^	26.1 ± 23.2 (n = 169)	35.6 ± 25.6 (n = 69)
Femoral neck	32.7 ± 23.4 (n = 94)	58.6 ± 33.7^§^ (n = 31)	24.7 ± 22.4 (n = 160)	33.2 ± 28.5 (n = 73)
Total hip	29.4 ± 22.5 (n = 130)	54.0 ± 34.6 (n = 47) ^װ^	25.4 ± 24.6 (n = 127)	35.4 ± 29.2 (n = 57)
Mid-radius	31.8 ± 25.4 (n = 87)	58.7 ± 37.1 (n = 32)**	25.1 ± 20.6 (n = 170)	38.2 ± 28.4 (n = 72)

*Back strength variable was log-transformed prior to testing.

†*P* = 0.041.

‡*P* = 0.002.

§*P* = 0.001.

װ*P* = 0.003.

¶*P* = 0.048.

**P = 0.001 (*t* test; T score>-1 vs T score≤-1, for the same sex).

Patients who received drugs that accelerate bone resorption (glucocorticoids, thyroxine) had significantly lower BMD (spine: *t* = 2.069, *P* = 0.39; femoral neck: *t* = 2.746, *P* = 0.006; total hip: *t* = 2.540, *P* = 0.011), but not muscle strength (*t* = 0.460, *P* = 0.646).

We used regression analysis to test the association between bone density and dynamometry, age, BMI, smoking, and glucocorticoid/thyroxine therapy. Before testing, back strength and smoking index variables were log-transformed. When controling for age and body mass index, the most significant predictor of BMD was glucocorticoid/thyroxine therapy at each region except at the mid-radius in women and at total hip in men. Muscle strength was significantly associated with spinal BMD, with total femur in men, and with mid-radius BMD in women (Table 4).

**Table 4:** Results of multiple regression analysis with bone mineral density (BMD) as dependent variable and different predictors

Predictors	BMD (g/cm^2^) (dependent variable), B (*P*)							
	spine		neck		total hip		mid-radius	
	women	men	women	men	women	men	women	men
Age (years)	-0.205 (0.271)	-0.247 (0.099)	-0.450 (0.015)	-0.226 (0.137)	-0.188 (0.210)	-0.193 (0.219)	-0.300 (0.079)	-0.014 (0.951)
Body mass index (kg/m^2^)	0.218 (0.191)	0.419 (0.011)	0.472 (0.004)	0.393 (0.018)	0.624 (<0.001)	0.394 (0.023)	0.274 (0.072)	0.329 (0.200)
Smoking (log)	-0.213 (0.208)	-0.144 (0.316)	-0.132 (0.408)	-0.162 (0.272)	-0.228 (0.096)	-0.172 (0.266)	-0.004 (0.973)	-0.201 (0.401)
Glucocorticoid and thyroxine therapy	-0.290 (0.097)	-0.660 (<0.001)	-0.062 (0.702)	-0.676 (<0.001)	0.119 (0.385)	-0.676 (<0.001)	-0.007 (0.962)	0.207 (0.381)
Muscle strength (log)	0.412 (0.027)	0.448 (0.010)	0.020 (0.908)	0.240 (0.120)	0.152 (0.318)	0.320 (0.041)	0.415 (0.020)	0.254 (0.299)
Adjusted R^2^ (P value)	0.237 (0.022)	0.644 (<0.001)	0.310 (0.006)	0.628 (<0.001)	0.510 (<0.001)	0.593 (0.001)	0.380 (0.001)	0.116 (0.251)

## Discussion

Our results showed that patients with hand and shoulder osteoarthritis had lower back strength than patients with osteoarthritis on other joints. Patients with inflammatory arthritis had the lowest bone density at the spine and femoral neck and they also had low back strength. Patients with rheumatic condition and with a low BMD also had lower back strength and that connection was more significant in men than in women.

The limitation of our study was a relatively small number of patients with inflammatory arthritis in comparison with patients with other rheumatic diagnoses. However, there were no differences in age and body mass index between them, so the major confounding factors were eliminated when comparing BMD and muscle strength between patients with inflammatory and non-inflammatory arthritis. Although significant, some reported correlation coefficients indicated weak or negligible correlation and should be considered with caution.

There are not many studies that analyzed the relationship between muscle strength and BMD in patients with rheumatic conditions. In patients with rheumatoid arthritis, Madsen found that femoral BMD was associated with quadriceps strength (4). Hakkinen et al showed that the decline of muscle strength in women with rheumatoid arthritis was observed earlier in the course of the disease than bone loss (3). Muscle strength was also pronouncedly reduced in patients with knee osteoarthritis, while BMD of the proximal tibia was not predictive of osteoarthritis symptoms (6).

In our study, correlation and regression analyses showed a significant association between muscle strength and BMD. Moreover, we found significantly lower muscle strength in patients who had a lower BMD. This is similar to the findings from most of other studies, which did not always include only rheumatic patients (9,12,20,21). Some of those studies measured grip strength (9-11,15), while others measured the strength at weight-bearing sites or back strength (4,13,14). However, they all showed either moderate or strong positive correlation between BMD and muscle strength. When considering rheumatic patients similar to those in our study, most of them had reduced muscle strength (3,22,23). There are only a few studies which have analyzed the connection between bone quality and muscle strength in patients with rheumatic conditions and in those studies, like in our study, bone density parameters were associated with muscle strength (3-5).Similar findings were presented in studies on bone density and muscle strength that comprised patients with some other diseases (24-26). The most significant predictor of bone density in our patients was steroid/thyroxine therapy, which is a well-known promoter of bone resorption (27,28). There are also studies which showed that steroid therapy can reduce muscle strength. Danneskiold-Samsoe and Grimby found that patients with rheumatoid arthritis receiving glucocorticoid agents had a 30% to 40% decrease in muscle strength and walking speeds compared with similar patients not receiving steroids (29).

Our patients with hand and shoulder osteoarthritis had the lowest back strength, which was probably due to the difficulty in performing dynamometry. Pulling the bar upwards puts the greatest load on the back and shoulder muscles. In our study group, the patients with shoulder osteoarthritis were usually partly or completely unable to perform that measurement and the dynamometry of zero pounds was most frequent in that group. Van Meteren et al also found that dynamometric measurements of both shoulders in 20 healthy participants were significantly different from the baseline measurements of 9 patients with shoulder disorders (20).

Our results did not show a significant site-specific relationship of muscle strength with osteoarthritis, because back strength was not lowest in patients with spinal osteoarthritis (cervico-brachial and lumbosacral syndrome). However, that site-specific relationship between bone density and osteoarthritis was more pronounced, because our patients with lumbosacral syndrome had a lower BMD than patients with hand, knee, or cervical spine osteoarthritis. Similar results were shown by Ock et al, who found a moderate correlation between hand-grip strength and hand BMD in healthy men (9). As we emphasized, there are no studies that explored that relationship specifically in patients with rheumatic conditions.

We conclude that there is a correlation between bone density and muscle strength in patients with rheumatic conditions. When we excluded patients with hand and shoulder rheumatic conditions, patients with inflammatory arthritis had the lowest muscle strength. Bone density was also lowest in patients with inflammatory arthritis. These results suggest that a concomitant decline in bone density and muscle strength is most pronounced in patients with inflammatory joint diseases compared with other rheumatic conditions.
